# Research capacity and culture in podiatry: early observations within Queensland Health

**DOI:** 10.1186/1757-1146-6-1

**Published:** 2013-01-09

**Authors:** Peter A Lazzarini, Julia Geraghty, Ewan M Kinnear, Mark Butterworth, Donna Ward

**Affiliations:** 1 Allied Health Research Collaborative, Metro North Health Service District, Queensland Health, Brisbane, Australia; 2 Department of Podiatry, Metro North Health Service District, Queensland Health, Brisbane, Australia; 3 School of Clinical Sciences, Queensland University of Technology, Brisbane, Australia; 4 Department of Allied Health Services, Metro North Health Service District, Queensland Health, Brisbane, Australia; 5 Department of Clinical Psychology & Neuropsychology, The Prince Charles Hospital, Queensland Health, Brisbane, Australia

**Keywords:** Podiatry, Research, Culture, Capacity, Australia

## Abstract

**Background:**

Research is a major driver of health care improvement and evidence-based practice is becoming the foundation of health care delivery. For health professions to develop within emerging models of health care delivery, it would seem imperative to develop and monitor the research capacity and evidence-based literacy of the health care workforce. This observational paper aims to report the research capacity levels of statewide populations of public-sector podiatrists at two different time points twelve-months apart.

**Methods:**

The Research Capacity & Culture (RCC) survey was electronically distributed to all Queensland Health (Australia) employed podiatrists in January 2011 (n = 58) and January 2012 (n = 60). The RCC is a validated tool designed to measure indicators of research skill in health professionals. Participants rate skill levels against each individual, team and organisation statement on a 10-point scale (one = lowest, ten = highest). Chi-squared and Mann Whitney U tests were used to determine any differences between the results of the two survey samples. A minimum significance of p < 0.05 was used throughout.

**Results:**

Thirty-seven (64%) podiatrists responded to the 2011 survey and 33 (55%) the 2012 survey. The 2011 survey respondents reported low skill levels (Median < 4) on most aspects of individual research aspects, except for their ability to locate and critically review research literature (Median > 6). Whereas, most reported their organisation’s skills to perform and support research at much higher levels (Median > 6). The 2012 survey respondents reported significantly higher skill ratings compared to the 2011 survey in individuals’ ability to secure research funding, submit ethics applications, and provide research advice, plus, in their organisation’s skills to support, fund, monitor, mentor and engage universities to partner their research (p < 0.05).

**Conclusions:**

This study appears to report the research capacity levels of the largest populations of podiatrists published. The 2011 survey findings indicate podiatrists have similarly low research capacity skill levels to those reported in the allied health literature. The 2012 survey, compared to the 2011 survey, suggests podiatrists perceived higher skills and support to initiate research in 2012. This improvement coincided with the implementation of research capacity building strategies.

## Background

Evidence-based practice is rapidly becoming the foundation of global clinical health care [1-3]. It involves the synthesis of research evidence to inform clinical practice [1-3]. Furthermore, new research is a major driver of global health care improvement [4,5]. The World Health Organisation has recently recognised that national health research plans should align with national health priorities, be multi-disciplinary and intersectoral in nature, and that research capacity should be strengthened in the public sector [5]. Australia’s National Health & Medical Research Council has stated that any new national strategic review of health research should plan for Australian health practitioners that are firmly embedded in evidence-based practice that has been developed from clinical research [6].

For a health profession to survive and prosper in a health care system that is focussed on evidence-based practice, it seems imperative for that profession to build its own research capacity and evidence-based literacy, particularly in the allied health professions [2,3,7,8]. However, it appears that the allied health professions are less developed than the medical and nursing professions in respect to explicitly building research capacity and addressing the future challenge of evidence-based practice [9-13]. Thus, it has been recognised that long-term strategies are needed to systematically build the research capacity of the diverse range of allied health professions, including podiatry [11,13,14]. A body of research has begun to focus on measuring the existing research capacity of allied health professions, whilst investigating strategies that may facilitate improving their overall research capacity [10-14].

Research capacity building has been described as the process of development which produces increased levels of skills to perform high quality research in individuals, teams or organisations [15]. Research capacity levels have historically been measured by traditional academic outputs including number of publications, citations, conference presentations, doctoral students, collaborators and grant funding [16]. However, over the last decade more contemporary research capacity tools measuring participants’ self-rated skills to carry out research have been developed; including skills in searching literature, accessing research infrastructure and support, writing research protocols, applying for funding, collecting data, analysing results and writing research manuscripts [10,17-19]. Allied health professions, although diverse, have been collectively rated as having low research skills or capacity [10,12,13]. In contrast, overall allied health professionals’ level of research interest appears to be much higher than their research skills [12,13].

The literature indicates a consistent set of motivators and barriers that influence research capacity levels in allied health professionals [13,20]. Motivators for professionals to undertake research have included: desire to increase skills, improve job satisfaction, advance career opportunities, address identified clinical problems, and engage with universities and research mentors [20]. Some of the barriers commonly identified included: lack of research time, funds, skills, backfill, research infrastructure, and that other work takes priority [20]. However, programs that have adopted strategies to address some of these barriers and enhance motivators have produced encouraging results in allied health professions, including demonstrated improvements in research capacity levels and traditional research outputs across various research experience levels [12,14,19,21,22]. These multi-faceted strategies commonly include specific allied health research training programs [14,19,22], available research funding and infrastructure support, [7,11,14,19,21], organisational and senior manager support [1,11,14,19,21], designated research coordinators and mentoring [7,14,19,21,23] and strategic research plans [5,8,11].

Podiatry has been identified as potentially having a comparatively high collective interest in doing research amongst the allied health disciplines [13,24]. Large surveys of the profession indicate that most podiatrists are very supportive of evidence-based practice and research, however, the majority desired more time, training and funding to implement research and evidence-based practice [24]. However, studies reporting the research capacity levels of small sub-groups of podiatrists (approximately five participants) suggest research skill levels are low in alignment with those reported in other allied health professions [13,19,21]. Similarly, studies have reported increases in the research capacity levels of small teams of podiatrists with the implementation of research capacity building strategies [19,21]. However, to the best of the authors’ knowledge, the research capacity levels of larger samples of podiatrists only have yet to be reported.

In 2008, Queensland Health recognised the importance of building research capacity within its allied health workforce by investing in a ‘Health practitioner research capacity building program’ in its 2007 enterprise bargaining agreement [25]. The package included creating 15 full time allied health research positions and allocating $400,000 in annual research grants by the end of the agreement [25]. The aims of the program and positions were “to build research capacity in the Health Practitioner workforce and facilitate the implementation of evidence-based clinical services” [25]. One of these positions was the 2011 establishment of a full-time (conjoint Queensland Health and Queensland University of Technology) podiatry research fellow position, that aimed to facilitate statewide Queensland Health clinical research capacity building in high risk foot care and podiatry [26].

With the introduction of designated allied health and podiatry research capacity building programs across Queensland Health it appeared an opportune time to measure and monitor the research capacity of podiatrists within Queensland Health. This observational paper aims to report the research capacity levels of statewide populations of public-sector podiatrists at two different time points twelve months apart.

## Methods

This study is part of a larger longitudinal observational study designed to monitor the annual cross-sectional research capacity levels of a population of Queensland Health podiatrists over four years. This paper reports on the first two of these annual cross-sectional surveys taken from a sample of convenience on both occasions. The Human Research Ethics Committee at The Prince Charles Hospital, Metro North Health Service District, Brisbane, Australia provided ethical approval for the study.

### Setting and participants

Eligible participants for each survey included all podiatrists employed by Queensland Health in the months of January 2011 or January 2012 as identified via the Queensland Health Podiatry Network database. The Queensland Health Podiatry Network is a formal network that represents all Queensland Health podiatrists. Queensland Health is the largest employer of publicly funded podiatrists in Queensland and employs approximately 10% of all Queensland registered podiatrists [27]. All participants resided in Queensland, Australia at the time of the surveys. Exclusion criteria included podiatrists not employed by Queensland Health at the time of the surveys and any podiatrists who were co-investigators of this study.

### Procedure

An invitation and electronic survey were sent via email to all eligible participants in the months of January 2011 (n = 58) and January 2012 (n = 60). The surveys remained open for 4 weeks on both occasions. Reminder emails were sent to all eligible participants at 2 weeks and 3 weeks following the initial emails to encourage completion. All eligible participants were advised that completion of the survey was voluntary and anonymous. The authors considered anonymity necessary to encourage full and open participation due to the relatively small number of Queensland Health podiatrists and the familiarisation this may bring to the authors. Participants consented to participating in the survey by ticking a box to confirm their consent.

### Survey instrument

The survey used on both occasions was the Research Capacity and Culture (RCC) tool [10]. The RCC tool is a valid and reliable survey tool designed to measure various indicators of research capacity and culture [10]. It was developed to accurately measure individual’s research skills, however, unlike other existing validated tools [18], it also measures participants perceptions of their team’s and organisation’s skills in research, or the holistic ‘research culture’ surrounding the individual [10]. It uses a series of statements designed to allow the respondent to rate their individual, their team’s and their organisation’s research skill level. Participants score each statement on a 10-point scale, with one being the lowest and ten being the highest skill level. Furthermore, the survey investigates participants perceived individual research barriers and motivators, plus, captures general demographic information [10].

### Statistical analysis

Data were analysed using SPSS 19.0 for Windows (SPSS Inc., Chicago, IL, USA). Descriptive statistics were used to display variable data using numbers and proportions for categorical data unless otherwise indicated. Chi-squared tests were used to test any differences between the 2011 and 2012 survey groups for dichotomous demographic, research activity, barrier and motivator variables. Whilst Mann Whitney U tests were used to test differences between the 2011 and 2012 survey groups for individual, team and organisation research capacity variables. The authors chose to analyse each 10-point scale item as ordinal categorical data to align with the categorical data parameters used by the RCC authors [10]. A minimum significance level of *p* < 0.05 was used throughout.

## Results

Thirty-seven (64%) eligible participants returned the 2011 survey. Thirty-three (55%) eligible participants returned the 2012 survey. Table 1 displays the general demographic details of respondents in both survey groups. Overall, the demographics were very similar for respondents in both survey groups, except the 2012 group had more participants that were permanent part-time employees and fewer with less than 2 years Queensland Health experience (p < 0.05). 

**Table 1:** **Demographic details of respondents**’ **to the 2011 and 2012 surveys**

		**2011 Survey**	**2012 Survey**	***p *Value**
Gender		*n* = 32 (%)	*n* = 32 (%)	
	Male	11 (34.4)	13 (40.6)	0.606
	Female	21 (65.6)	19 (59.4)	0.606
Year born		*n* = 33 (%)	*n* = 32 (%)	
	Before 1946	0	0	NA
	Between 1946-1951	0	0	NA
	Between 1952-1956	3 (9.1)	1 (3.0)	NA
	Between 1957-1962	1 (3.0)	1 (3.0)	NA
	Between 1963-1967	5 (15.2)	4 (12.5)	0.757
	Between 1968-1972	5 (15.2)	6 (18.8)	0.700
	Between 1973-1978	6 (18.2)	11 (34.4)	0.138
	Between 1979-1983	5 (15.2)	3 (9.4)	0.479
	Between 1984-1988	8 (24.20)	6 (18.8)	0.590
	After 1988	0	0	NA
Service		*n* = 33 (%)	*n* = 32 (%)	
	Hospital	16 (48.5)	14 (43.8)	0.702
	Clinical	5 (15.2)	4 (12.5)	0.757
	Sub-Acute Facility	3 (9.1)	2 (6.3)	0.667
	Community Health Centre	16 (48.5)	22 (68.8)	0.097
	Nursing Home	2 (6.1)	1 (3.1)	0.573
	Management	3 (9.1)	1 (3.1)	0.317
Current classification level		*n* = 33 (%)	*n* = 32 (%)	
	HP 1-2	0	0	NA
	HP 3	8 (24.2)	8 (25.0)	0.944
	HP 4	15 (45.5)	16 (50.0)	0.714
	HP 5	7 (21.2)	6 (18.8)	0.804
	HP 6	3 (9.1)	2 (6.3)	NA
	HP 7 or above	0	0	NA
Years experience in Podiatry		*n* = 33 (%)	*n* = 30 (%)	
	Less than 2 years	5 (15.2)	2 (6.7)	0.285
	2-5 years	6 (18.2)	6 (20.0)	0.854
	6-10 years	6 (18.2)	7 (23.3)	0.614
	11-15 years	10 (30.3)	9 (30.0)	0.979
	16-20 years	1 (3.0)	3 (10.0)	NA
	20+ years	5 (15.2)	3 (10.0)	0.540
Years experience in QH as a Podiatrist		*n* = 33 (%)	*n* = 30 (%)	
	Less than 2 years	15 (45.5)	7 (23.3)	0.066
	2-5 years	9 (27.3)	15 (50.0)	0.064
	6-10 years	7 (21.2)	3 (10.0)	0.224
	11-15 years	1 (3.0)	4 (13.3)	NA
	16-20 years	1 (3.0)	0	NA
	20+ years	0	1 (3.3)	NA
Years worked in QH in any capacity		*n* = 33 (%)	*n* = 32 (%)	
	Less than 2 years	13 (39.4)	5 (15.6)	0.032
	2-5 years	10 (30.3)	16 (50.0)	0.105
	6-10 years	7 (21.2)	5 (15.6)	0.562
	11-15 years	1 (3.0)	4 (12.5)	NA
	16-20 years	2 (6.1)	0	NA
	20+ years	0	2 (6.3)	NA
Current employment status		*n* = 33 (%)	*n* = 32 (%)	
	Permanent full-time	24 (72.7)	17 (53.1)	0.102
	Permanent part-time	1 (3.0)	6 (18.8)	0.041
	Temporary full-time	6 (18.2)	8 (25.0)	0.504
	Temporary part-time	1 (3.0)	1 (3.1)	NA
	Temporary casual	0	0	NA
Future in QH		*n* = 33 (%)	*n* = 32 (%)	
	Less than 2 years	0	1 (3.1)	NA
	2-4 years	3 (9.1)	2 (6.3)	NA
	5-9 years	7 (21.2)	6 (18.8)	0.804
	10+ years	16 (48.5)	11 (34.4)	0.249
	Not sure	7 (21.2)	12 (37.5)	0.149

Tables 2, 3, 4 display the median ratings and interquartile range results with respect to respondents’ individual, team and organisation research skills in the 2011 and 2012 surveys. Table 2 demonstrates that participants’ rated their individual skill level high enough to find, store and critically review relevant literature in both surveys (Median < 7). However, the 2011 survey respondents’ rated that they lacked adequate skills to undertake any other aspects of research (Median < 4). In the 2012 survey a number of items reported significantly higher levels of skill than in 2011; including a higher reported skill level to secure research funding; submit ethics applications; and provide advice to less experienced researchers (p < 0.05). 

**Table 2:** **Individual research skill ratings **– **medians and interquartile range **(**M **(**IQR**))

		**2011 Survey**		**2012 Survey**		
**Individual (self) research skill statement**		***n***	**M(IQR)**	***n***	**M(IQR)**	***p *Value**
1	finding relevant literature	34	7 (4 – 8)	32	7 (5.25 – 8)	0.366
2	critically reviewing the literature	33	6 (4 – 8)	32	7 (5 – 8)	0.170
3	using a computer referencing system (e.g., Endnote)	33	6 (2 – 7)	32	5 (2.25 – 7)	0.761
4	writing a research protocol	33	3 (2 – 6)	31	5 (3 – 7)	0.108
5	securing research funding	32	2 (1 – 3.75)	31	4 (2 – 5)	0.024
6	submitting an ethics application	33	2 (1 – 3.5)	31	4 (2 – 6)	0.034
7	designing questionnaires	32	3 (2 – 5.75)	31	4 (3 – 7)	0.099
8	collecting data (e.g., surveys, interviews)	33	4 (2 – 6.5)	31	5 (3 – 6)	0.128
9	using computer data management systems	32	3 (2 – 6)	31	5 (3 – 7)	0.271
10	analysing qualitative research data	33	3 (2 – 5)	31	4 (3 – 6)	0.698
11	analysing quantitative research data	32	3.5 (2 – 5)	31	5 (3 – 6)	0.377
12	writing a research report	33	3 (2 – 5.5)	31	5 (3 – 7)	0.074
13	writing for publication in peer-reviewed journals	33	2 (2 – 5)	31	4 (2 – 6)	0.153
14	providing advice to less experienced researchers	33	2 (1–4)	31	4 (2 – 6)	0.043

Note: Missing data and unsure responses have been removed from above sample.

**Table 3:** **Team research skill ratings **– **medians and interquartile range **(**M **(**IQR**))

		**2011 Survey**		**2012 Survey**		
**Team research skill statement**		***n***	**M(IQR)**	***n***	**Median(IQR)**	***p *Value**
1	has adequate resources to support staff research training	27	3 (2 – 5)	32	6 (3.25 – 7.75)	<0.001
2	has funds, equipment or admin to support research activities	27	3 (2 – 5)	27	5 (2 – 6)	0.029
3	does team level planning for research development	29	3 (1.5 – 6)	29	4 (2 – 6.5)	0.178
4	insures staff involvement in developing that plan	29	3 (2 – 6)	29	4 (2.5 – 8)	0.067
5	has team leaders that support research	33	7 (3.5 – 8)	32	7 (5 – 9)	0.264
6	provides opportunities to get involved in research	30	5 (1 – 7)	30	5 (3.75 – 8)	0.151
7	does planning that is guided by evidence	32	7 (3.5 – 9)	30	7 (5 – 9)	0.932
8	has consumer involvement in research activities/planning	23	3 (1 – 5)	28	4 (3 – 5.75)	0.093
9	has applied for external funding for research	20	2 (1 – 5)	26	3 (1 – 8.25)	0.261
10	has easy access to literature searching and article retrieval	34	8 (4.75 – 9)	32	8 (5.25 – 9)	0.672
11	conducts research activities relevant to practice	31	6 (3 – 8)	31	7 (3 – 9)	0.382
12	support applications for research scholarships/degrees	30	7 (2.75 – 9)	31	7 (5 – 9)	0.323
13	has mechanisms to monitor research quality	17	3 (1 – 5.5)	26	5 (2.75 – 9)	0.033
14	has identified experts accessible for research advice	26	6.5 (2.75 – 8)	30	8.5 (4.75 – 9)	0.035
15	disseminates research results at research forums/seminars	28	5.5 (2 – 8)	30	4.5 (3 – 9)	0.919
16	supports a multi-disciplinary approach to research	27	7 (4 – 8)	30	7 (4 – 9)	0.955
17	has incentives and support for mentoring activities	27	6 (3 – 7)	30	7 (3 – 9)	0.204
18	has external partners (e.g., universities) engaged in research	24	4.5 (2 – 7.75)	26	5.5 (2.75 – 9)	0.384
19	has software available to support research activities	19	4 (1 – 5)	23	4 (2 – 7)	0.275
20	supports peer reviewed publication of research	--	--	29	7 (4 – 9)	--

Note: Missing data and unsure responses have been removed from above sample.

**Table 4:** **Organisation research skill ratings **– **medians and interquartile range **(**M **(**IQR**))

		**2011 Survey**		**2012 Survey**		
**Organisation research skill statement**		***n***	**M(IQR)**	***n***	**M(IQR)**	***p *Value**
1	has adequate resources to support staff research training	33	6 (3 – 7.5)	32	7 (5.25 – 8.75)	0.047
2	has funds, equipment or admin to support research activities	32	4 (3 – 6)	30	6.5 (4 – 7.25)	0.007
3	has a plan or policy for research development	33	6 (3 – 7.5)	27	7 (6 – 8)	0.079
4	has easy access to literature searching and article retrieval	37	8 (6.5 – 9)	33	8 (6 – 9)	0.986
5	has senior managers that support research	36	8 (5 – 9)	30	7 (6 – 9)	0.845
6	ensures staff career pathways are available in research	36	6.5 (4 – 8)	29	6 (4.5 – 8)	0.889
7	ensures organisation planning is guided by evidence	35	7 (5 – 9)	33	7 (5.5 – 9)	0.550
8	has consumers involved in research	26	5 (3 – 6.25)	28	6.5 (4 – 8)	0.057
9	accesses external funding for research	25	6 (3 – 8)	28	7.5 (5 – 8)	0.098
10	promotes clinical practice based on evidence	37	8 (7 – 9.5)	32	9 (8 – 9)	0.600
11	encourages research activities relevant to practice	31	8 (4 – 9)	31	8 (7 – 8)	0.570
12	has software programs for analysing research data	19	5 (1 – 8)	21	7 (3.5 – 8.5)	0.093
13	has mechanisms to monitor research quality	15	4 (2 – 8)	26	7 (4.27 – 9)	0.022
14	has identified experts accessible for research advices	28	7 (5 – 8)	30	8 (7 – 9.25)	0.041
15	supports a multi-disciplinary approach to research	33	7 (5 – 9)	27	8 (6 – 9)	0.119
16	has regular forums/bulletins to present research	31	6 (4 – 8)	29	7 (5 – 8)	0.478
17	engages external partners (e.g., universities) in research	28	6.5 (5 – 8)	30	8.5 (7 – 9)	0.004
18	supports the peer-reviewed publication of research	27	7 (4 – 9)	28	8 (7 – 9)	0.085
19	requires ethics approval for research activities	31	9 (7 – 10)	29	10 (8 – 10)	0.272
20	supports applications for research scholarships/degrees	--	--	28	8 (7 – 9)	--

Note: Missing data and unsure responses have been removed from above sample.

Table 3 reveals that participants in both survey groups rated their clinical team had team leaders that supported research, had team plans that were guided by evidence, supported multi-disciplinary approaches to research, had easy access to literature searching and article retrieval and were supportive of their applications for research scholarships or degrees (Median > 7). However, the 2011 survey respondents’ rated that their team did not have adequate research skill to satisfactorily perform the majority of research activities (Median < 5). In the 2012 survey, respondents’ reported significantly higher levels of team skills than in 2011; including having adequate resources to support staff research training; funds, equipment or administration to support research activities; mechanisms to monitor research quality; and identified experts accessible for research advice (p < 0.05).

Table 4 displays that participants in both survey groups rated their organisation’s research skill level to be adequate-to-high enough to perform nearly all identified aspects of research (Median > 5). In the 2012 survey, respondents’ reported significantly higher levels of organisational research skill than in 2011; including having adequate resources to support staff research training; funds, equipment or admin to support research activities; mechanisms to monitor research quality; identified experts accessible for research advice; and engagement of external partners (e.g. universities) in research in 2012 (p < 0.05).

Lastly, trends in higher ratings in the 2012 survey compared to the 2011 survey were evident for individual’s skill in writing a research report; team’s ensuring staff were involved in developing research plans; and organisation’s having a plan for research development and consumer involvement (p < 0.08) (Tables 2, 3, 4). No significant lower ratings were noted in the 2012 survey compared to the 2011 survey across Tables 2, 3, 4 (p < 0.05).

Table 5 illustrates the current research activities that individual respondents were involved in at the time of the 2011 and 2012 surveys. No significant differences were noted between the research activities reported by respondents in the 2011 and 2012 surveys; however, a trend in higher numbers of research protocols written was evident in the 2012 survey compared with the 2011 survey (p < 0.08). 

**Table 5:** **Individual **(**self**) **current research activities **– **numbers and **% (**n **(%))

**Research activities**		**2011 Survey*n *(%) =** **34(100)**	**2012 Survey*n *(%) =** **32(100)**	***p *Value**
1	Writing a research protocol	2 (5.9)	7 (21.9)	0.058
2	Submitting an ethics application	1 (2.9)	4 (12.5)	NA
3	Collecting data (e.g., surveys, interviews)	11 (32.4)	9 (28.1)	0.709
4	Analysing qualitative research data	1 (2.9)	1 (3.1)	NA
5	Analysing quantitative research data	4 (11.8)	4 (12.5)	NA
6	Writing for publication in a peer-reviewed journal	4 (11.8)	3 (9.4)	NA
7	Applying for research funding	2 (5.9)	6 (18.8)	0.109
8	No research activities	17 (50.0)	18 (56.3)	0.611

NA = Not applicable to test as the assumption of Chi-Squared test is violated as the 2 cells had expected counts of < 5.

Table 6 and 7 display the main motivators and barriers to participants performing research. The 2011 survey identified the top five motivators to perform research were: to develop research skills; increase job satisfaction; keep at the cutting edge of practice; career advancement; and identifying a problem that needed changing. The top five barriers to performing research identified in the 2011 survey were other work roles taking priority; lack of time for research; lack of suitable backfill; lack of skills in research; and general clinical isolation. The 2012 survey displayed significant differences compared to the 2011 survey in scores related to keeping at the cutting edge of practice, other work roles take priority, lack of skills in research and isolation (p < 0.05). 

**Table 6:** **Individual **(**self**) **research motivators **– **numbers and **% (**n **(%))

**Research motivators**		**2011 Surveyn(%) =** **34(100)**	**2012 Surveyn(%) =** **32(100)**	***p *Value**
1	To develop skills	31 (91.2)	28 (90.3)	0.905
2	Increased job satisfaction	27 (79.4)	22 (71.0)	0.430
3	Keeping at the cutting edge of practice	22 (64.7)	11 (35.5)	0.019
4	Career advancement	20 (58.8)	15 (48.4)	0.399
5	Problem identified that needs changing	16 (47.1)	9 (29.0)	0.136
6	Increased credibility	14 (41.2)	11 (35.5)	0.638
7	To keep the brain stimulated	14 (41.2)	9 (29.0)	0.306
8	Opportunities to participate on own level	10 (29.4)	6 (19.4)	0.347
9	Mentors available to supervise	10 (29.4)	10 (32.3)	0.804
10	Colleagues doing research	9 (26.5)	7 (22.6)	0.716
11	Desire to prove a theory/hunch	8 (23.5)	13 (41.9)	0.113
12	Research encouraged by managers	7 (20.6)	5 (16.1)	0.643
13	Grant funds	6 (17.6)	5 (16.1)	0.870
14	Study or research scholarships available	6 (17.6)	5 (16.1)	0.870
15	Dedicated time for research	5 (14.7)	6 (19.4)	0.618
16	Links to universities	3 (8.8)	8 (25.8)	0.068
17	Forms part of post-graduate study	3 (8.8)	6 (19.4)	0.220
18	Research written into role description	2 (5.9)	3 (9.7)	NA
19	No motivators	0 (0)	0 (0)	NA

NA = Not applicable to test as the assumption of Chi-Squared test is violated as the 2 cells had expected counts of < 5.

**Table 7:** **Individual **(**self**) **research barriers **– **numbers and **% (**n **(%))

**Research barriers**		**2011 Surveyn(%) =** **34(100)**	**2012 Survey n(%) =** **32(100)**	***p *Value**
1	Other work roles takes priority	29 (85.3)	20 (62.5)	0.034
2	Lack of time for research	27 (79.4)	23 (71.9)	0.475
3	Lack of suitable backfill	19 (55.9)	14 (43.8)	0.325
4	Lack of skills in research	18 (52.9)	9 (28.1)	0.040
5	Isolation	16 (47.1)	8 (25.0)	0.063
6	Lack of administrative support	15 (44.1)	11 (34.4)	0.418
7	Lack of funds for research	14 (41.2)	10 (31.3)	0.402
8	Desire for work/life balance	12 (35.3)	10 (31.3)	0.728
9	Lack of access to equipment for research	11 (32.4)	7 (21.9)	0.339
10	Lack of software for research	10 (29.4)	6 (18.8)	0.312
11	Intimidated by research language	10 (29.4)	8 (25.0)	0.688
12	Lack of a coordinated approach to research	9 (26.5)	5 (15.6)	0.281
13	Other personal commitments	9 (26.5)	7 (21.9)	0.663
14	Lack of support from management	7 (20.6)	7 (21.9)	0.898
15	Intimidated by fear of getting it wrong	5 (14.7)	6 (18.8)	0.660
16	Lack of library/internet access	3 (8.8)	2 (6.2)	NA
17	No interest in research	1 (2.9)	2 (6.2)	NA
18	No barriers	1 (2.9)	1 (3.1)	NA

NA = Not applicable to test as the assumption of Chi-Squared test is violated as the 2 cells had expected counts of < 5.

## Discussion

This paper appears to report the research capacity levels of the largest samples of podiatrists published. It also appears to be the first paper to use validated contemporary measures to investigate podiatrists’ perceptions of their individual, team and organisational research capacity levels. The findings indicate that podiatrists rated their overall individual skills at performing most aspects of research at a low level. In contrast, podiatrists rated the overall research skills and culture provided by their organisation to be of a high level if they chose to initiate research. Interestingly, the 2012 survey results, compared to those results taken twelve months earlier in the 2011 survey, indicated significantly higher skill levels on a range of individual, team and organisational research aspects required to initiate research projects.

Overall, the 2011 survey findings suggest podiatrists had the individual skills to search and appraise relevant literature, however, lacked the skills to then initiate and perform a research project [10,13]. These findings compared nearly equivalently to those found in similar populations of allied health practitioners [10,13] and were similar to those found in interdisciplinary populations involving medical, nursing and allied health clinicians [12,28]. However, perhaps understandably these individual research skill findings were lower than when compared to populations already participating or leading research [12].

Organisational and team skills to support and perform research, or the ‘research culture’, have been identified as having a large influence on individual health professionals’ skills and confidence to plan and implement clinical research [1,7,11,14,19]. Organisational research culture in particular was rated highly in both surveys, which suggests podiatrists felt Queensland Health would encourage and support their research aspirations. Interestingly, the results of both surveys in this study were similar to another similar study indicating that the podiatry population consistently perceived their teams and organisation to have higher research skills than their individual skills [10]. However, this paper reports consistently higher team and organisation skill levels than that of the other study investigating a very similar population of Queensland Health allied health practitioners [10]. This may be an indication of the pre-existing research culture of the podiatry profession, or perhaps, it may have been that the early generic Queensland Health strategies of the ‘health practitioner research capacity building program’ had already been perceived by podiatrists in this study.

Of particular note in the organisational findings were the very high levels that podiatrists perceived their organisation was promoting clinical practice based on evidence and research activities relevant to practice [11]. These findings seem to be higher than those reported in other Queensland Health allied health populations [10]. This may align with podiatrists’ reported interest in evidenced-based practice and research [13,24] and previously reported strategies in this particular population to promote and research the clinical outcomes of clinical practice based on evidence [29-32]. Furthermore, the findings of both surveys indicated that only a very low proportion of participants reported a lack of research interest as a barrier compared to previous similar studies [13,20]; conversely, this would suggest a very high proportion of podiatry participants were interested in doing research [12,13,20]. These findings concurred with other similar studies indicating that podiatrists have a comparatively high interest in doing research within a population of allied health practitioners that already report high levels of research interest [12,13,20]. However, this could be attributed to a respondent bias of those interested in research responding to these surveys and those not interested declining, thus, over-inflating the level of research interest.

The barriers to performing research in both surveys were comparable to those reported in similar allied health populations [7,20]. Most perceived the biggest barriers to be that other work roles take priority, and a lack of time, skills and suitable backfill are problems in performing research [7,19,20]. Interestingly, isolation was also identified as a major barrier in the initial 2011 survey, which wasn’t identified in a study investigating a similar population [20]. This may be the result of the Queensland Health podiatry population investigated in this study residing in diverse locations across Queensland rather than in the previously reported metropolitan-centric locations [20], and that the podiatry profession makes up comparatively small numbers compared to many other allied health professions [33]. However, the 2012 survey displayed an encouraging decreasing trend in the magnitude of isolation compared to the 2011 survey as a perceived barrier to performing research. This in turn may suggest that podiatrists in the second survey perceived more support to perform research in the 2012 survey, and thus, potentially less isolated than respondents felt in 2011.

Research motivators reported in the surveys of this study also closely mirrored those found in similar populations, and included the ability to develop skills, increase job satisfaction, career advancement and identify problems that need changing [19,20]. Interestingly, podiatrists perceived keeping at the cutting edge of practice and increased credibility as two high motivators that did not rate highly in other health professional populations [20]. It could be hypothesised, similarly to isolation, that as a comparatively small profession, there may be a sense of need to prove the credibility of the professions’ performance to patients and other health professionals that is not perceived necessary in other larger professions [34].

There appear to be a number of encouraging changes in the level of respondents’ perceived research skills in the 2012 survey compared with that of the 2011 survey. Overall, it would appear that skills reported in 2012 were higher in research activities that are important in the initiation of a research project. These activities included having the skills to secure research funding, submit an ethics application, provide advice to less experienced researchers, plus, a trend in increased skill levels to write a research report. This skill improvement also seems to have translated into a trend of performing the writing of more research protocols in 2012 compared with the 2011 survey. Furthermore, similar trends in higher reported skills to initiate research was perceived in regards to team and organisational skills and support in the 2012 survey, including the organisation providing adequate research training, infrastructure, expertise, planning, mechanisms to monitor quality research and promoting collaboration with universities. Encouragingly, there appears to be no diminishing of research skill, capacity or culture in 2012.

Although all higher research skill levels reported in the 2012 survey were in the main compared with very low skill levels reported in the 2011 survey, it is nonetheless a promising early trend. The higher levels reported in 2012 may be related to a host of possible factors. These included potential respondent bias from those participating being those most involved in research, testing bias from participants learning from their responses to the first survey, maturation bias from participants being older and more experienced at the second survey, or possibly the background generic Queensland Health research capacity building strategies had been improving research capacity since 2008. Furthermore, it could be speculated that the changes coincided with the introduction of a number of specific podiatry-related best practice research capacity building strategies implemented across Queensland Health between the 2011 and 2012 surveys. These strategies included the appointment of a statewide podiatry research fellow [26], collaborations with other research fellows and senior management [1,11,14,19,21], a statewide strategic research plan [5,8,11,19], research training and workshops [14,19,22], grant funding [7,11,14,19,21], research mentoring and forums [7,14,19,21,23] and peer-reviewed publications [19]. Thus, it could be postulated that these strategies, previously reported to improve interdisciplinary research capacity [1,11,14,19,21], may have influenced some of the changes in skill levels reported between the 2011 and 2012 surveys. However, due to the observational nature of the study it is not possible to definitively link any factor in a cause-and-effect relationship with the reported results.

Finally, the demographic make-up of the two samples appear to align with the demographic makeup of that reported for the broader Australian podiatry population; including participants having a median age bracket of 36 – 40 years, approximately 40% being male and practicing across diverse geographical locations [27]. This may assist with the generalisability of the results of this study, however, this study only reported on podiatrists that were publicly employed. Publicly funded podiatrists in Australia make up only a small proportion of the total numbers of podiatrists employed [27], and thus, this study may not reflect the perceptions of other podiatry sectors. Furthermore, the similarity in demographics of both samples suggests some participants completed both surveys.

### Limitations

There have been a number of limitations already outlined in this study; including respondent bias, testing bias, maturation bias, inability to generalise results to other non-public podiatry sectors, and the observational nature of this study being unable to ascertain a cause-and-effect relationship to any possible intervening factors to this study. However, other limitations also exist. Firstly, even though the podiatry sample sizes of both surveys were the largest reported in the literature and adequate in overall numbers for appropriate statistical testing (n > 30), they were still considerably smaller than other comparable allied health studies. Furthermore, although the overall sample sizes were adequate for appropriate statistical testing, any subgroup analyses as per other studies, were not attempted due to the very high possibility for small subgroup numbers (n < 30) and the risk of committing a Type I statistical error. Secondly, this study did not have a control group; however, this may be partially addressed by comparing our results with those previously published for a very similar Queensland Health allied health population [10,20].

Thirdly, it appears some participants completed both surveys, thus, it may have been preferable to use only matched respondents and paired statistical tests. However, the authors originally estimated this methodology would have resulted in a very small sample size and a substantial risk of a Type I statistical error; the authors calculated a final paired sample of just 15 participants assuming a 50% initial response rate and a 15% annual attrition rate over four years. Conversely, the authors’ use of unpaired statistical tests, in a population that may contain some of the same participants, increased the risk of committing a Type II statistical error of finding no differences when their actual were statistical differences. The authors decided to accept the risk of a Type II error, and thus, some of the trend results (p < 0.08) of this paper may have reached significance with a slightly different methodology and analysis.

Lastly, this study appears to be the first to use the RCC since it was validated. The authors found the survey to be efficient and effective to implement and analyse for the purposes of measuring holistic research capacity. In particular it’s use of ten-point scales seemed to allow for more defined reporting and analysis of the magnitude of research skills, in comparison to previously published surveys [18,19]. Furthermore, it would appear that participants found it no less attractive to complete than other similar surveys, with all reporting response rates in excess of 50% [13,18], and, the high completion rate of all 54 research skill items on both surveys in this study. However, one area that may be problematic and need further definition was the domain of teams, particularly in a population that covers a large diverse geographic region and contains many different sub-organisations. Anecdotally, the definition of team appeared to differ to different participants and this may distort the reliability of team results.

Whilst these limitations need to be taken into account, these findings are promising in the context of health professions needing to measure and transition their cultures to that of an integrated evidence-based practice and research culture to survive [2,3,7,8]. These early results support the continuation of a larger longitudinal observational study to monitor further changes to research capacity levels in this podiatry population over time. However, it is recommended that larger experimental studies investigating the impact of multi-faceted research capacity building interventions on the research capacity levels and outputs of a larger multi-sectoral podiatry population are implemented. Furthermore, the authors support the recommendations of the UK Society of Chiropodists and Podiatrists, to optimise the existing high podiatry interest in evidence-based practice and research, by implementing nationally coordinated multi-faceted strategies to build research capacity for the future benefit of the podiatry profession and its patients [8,35-37].

## Conclusions

This study appears to report the research capacity of the largest population of podiatrists published. The findings are consistent with existing literature indicating podiatry practitioners are skilled at searching and reviewing relevant literature, however, their skills in performing other research activities are low. The improved reported research skill levels of podiatrists’ to perform early research activities in the second survey that coincided with the introduction of multi-faceted strategies to build research capacity shows promise. Furthermore, the comparatively high reported podiatry research interest and skills in employing evidenced-based practice is encouraging. It is recommended that research capacity levels of this population of podiatrists is investigated over the longer term and that the podiatry profession consider national implementation of research capacity measures and interventions if the profession is to prosper in the evidence-based practice world in which we live.

## Competing interests

PAL is the conjoint Queensland Health/Queensland University of Technology Research Fellow (Podiatry) identified in the manuscript. Otherwise, the authors have no relevant conflict of interest to disclose.

## Authors’ contributions

PAL and JG conceived, designed, researched data, contributed to discussion, wrote and reviewed/edited the manuscript. EMK and MB contributed to discussion and reviewed/edited the manuscript. DW conceived, contributed to discussion and reviewed/edited the manuscript. All authors read and approved the final manuscript.
